# Targeting miR-10b in Breast Cancer Bone Colonization Model Using Image-Guided Nucleic Acid-Based Therapeutics

**DOI:** 10.3390/cancers18172731

**Published:** 2026-08-23

**Authors:** Sujan K. Mondal, Elizabeth Kenyon, Bryan Doyun Kim, Zdravka Medarova, Anna Moore

**Affiliations:** 1 Precision Health Program, Michigan State University, 766 Service Road, East Lansing, MI 48824, USA; mondalsu@msu.edu (S.K.M.); kenyonel@msu.edu (E.K.); kimbrya1@msu.edu (B.D.K.); 2 Department of Radiology, College of Human Medicine, Michigan State University, 846 Service Road, East Lansing, MI 48824, USA; 3 TransCode Therapeutics, Inc., 400 Trade Center, Suite 5900, Woburn, MA 01801, USA; zdravka.medarova@transcodetherapeutics.com

**Keywords:** breast cancer metastasis, microRNA-10b, anti-sense oligonucleotides, nanoparticles, in vivo imaging

## Abstract

**Background/Objectives:** Breast cancer is the most frequently diagnosed cancer among women worldwide and a leading cause of cancer-related death. Despite the use of bisphosphonates, RANKL inhibitors, chemotherapy, and available endocrine therapies, the five-year survival rate for patients with bone metastases remains approximately 20–30%, underscoring the urgent need for novel, metastasis-specific therapeutic strategies. Recent studies demonstrated that miR-10b can serve as an attractive therapeutic target for the treatment of metastatic breast cancer, particularly in the context of bone metastasis. This study aimed to test antimir-10b therapeutics in a mouse model of breast cancer bone metastasis. **Methods:** We utilized a previously developed dextran-coated iron oxide nanoparticle-based platform for delivery of antisense anti-miR-10b oligonucleotides to bone metastases. The magnetic properties of the nanoparticles allowed for in vivo imaging of therapeutic delivery to metastatic tumors. **Results:** We showed the delivery of the therapeutics in the bone colonization model by in vivo imaging as well as significant survival benefits in injected animals. There was a significant inhibition of miR-10b following treatment that resulted in significant upregulation of the downstream target HOXD10 in vitro and a similar trend in vivo. Repeated dosing of the therapeutics was well tolerated, and no systemic toxicity was observed, supporting the safety profile of this approach. **Conclusions:** Collectively, these studies demonstrated that targeting miR-10b using an image-guided anti-miR-10b nanotherapeutic represents a promising and translatable strategy for targeting breast cancer bone metastases.

## Simple Summary

The majority of cancer-related deaths result from metastatic disease rather than the primary tumor. Bone is the predominant site of distant metastasis in breast cancer and represents one of the greatest clinical challenges, affecting up to 70% of patients with advanced disease. Studies from several laboratories, including our own, have demonstrated that small molecules within cancer cells called microRNAs (miRs) play critical roles in the initiation and progression of metastasis by regulating tumor cell invasion and growth. Therefore, therapeutic inhibition of metastasis-promoting miRNAs could lead to an effective and persistent regression and possibly elimination of metastases in breast cancer patients. In this study, we demonstrated the therapeutic efficacy of targeting miR-10b in a preclinical breast cancer bone colonization model. We hope that these results will facilitate the translation of miR-10b-targeted therapies into clinic and ultimately lead to more effective treatment options for patients with metastatic breast cancer.

## 1. Introduction

Breast cancer is the most frequently diagnosed cancer among women worldwide and a leading cause of cancer-related death [1,2]. While advances in early detection and systemic therapies have significantly improved outcomes for patients with localized disease, metastatic breast cancer remains mostly incurable [3,4,5]. Bone is the predominant site of distant metastasis in breast cancer and represents one of the greatest clinical challenges, affecting up to 70% of patients with advanced disease [6,7,8,9,10]. Breast cancer bone metastasis is associated with severe skeletal-related events, including bone fractures, spinal cord compression, hypercalcemia, and intractable pain, which collectively result in substantial morbidity and diminished quality of life [6]. Despite the use of bisphosphonates, RANKL inhibitors, chemotherapy, and endocrine therapies to manage skeletal complications and tumor burden, the five-year survival rate for patients with bone metastases remains approximately 20–30% [11,12], underscoring the urgent need for novel, metastasis-specific therapeutic strategies.

The development and progression of breast cancer bone metastases are maintained by complex interactions of tumor cells with the bone microenvironment. Increasing evidence suggests that these metastatic processes are tightly regulated by non-coding RNAs [13,14], particularly microRNAs (miRNAs) [15,16], which function as post-transcriptional regulators of gene expression [17]. Dysregulation of specific miRNAs has been implicated in virtually all stages of the metastatic process, including epithelial-to-mesenchymal transition, invasion, intravasation, survival in circulation, and colonization of distant organs [18,19,20,21].

MicroRNA-10b (miR-10b) has emerged as a key player of metastatic progression in breast cancer. Increased miR-10b expression is strongly correlated with tumor invasiveness, metastatic burden, and poor clinical outcomes [15,22,23]. Mechanistically, miR-10b promotes metastasis by suppressing key tumor suppressors and regulators of cell motility, thereby enhancing invasion, migration, and survival of metastatic cells [15]. These characteristics make miR-10b an attractive therapeutic target for the treatment of metastatic breast cancer, particularly in the context of bone metastasis.

Despite the strong therapeutic justification for targeting miR-10b, clinical translation of miR-10b-targeting therapies has been limited partly by delivery-related challenges [24]. Antisense oligonucleotides targeting miRNAs are inherently not stable in circulation, highly susceptible to nuclease degradation, and often exhibit poor cellular uptake [25,26]. Additionally, selective accumulation of anti-miR therapeutics at metastatic sites, particularly within the bone microenvironment, while minimizing off-target distribution and systemic toxicity remains a major challenge [27]. The bone metastatic niche presents significant barriers, including restricted vascular access, high interstitial pressure, and a dense mineralized matrix further limiting effective drug penetration [6,7,28]. These challenges highlight the need for robust delivery platforms that can protect nucleic acid cargo, promote tumor-selective accumulation, and enable assessment of in vivo accumulation.

To address these limitations of oligonucleotide delivery, we have previously developed a dextran-coated iron oxide nanoparticle-based platform for delivery of antisense anti-miR-10b oligonucleotides (MN-anti-miR10b). In prior studies, MN-anti-miR10b demonstrated efficient delivery to primary and metastatic tumor sites in small and large animals, potent inhibition of miR-10b activity, and significant suppression of metastatic growth in vivo, particularly in lymph node metastasis models of breast cancer, without inducing systemic toxicity [23,29,30,31,32,33]. In addition to favorable biocompatibility and pharmacokinetics, iron oxide nanoparticles are intrinsically superparamagnetic, enabling delivery monitoring by noninvasive magnetic resonance imaging (MRI). When combined with fluorescent labeling, this platform allows multimodal imaging and real-time assessment of nanoparticle biodistribution and tumor targeting [29,30,31,32,34,35]. Developed under the commercial name TTX-MC138, this therapeutic has completed the IND-enabling package, including studies of stability, pharmacokinetics, biodistribution, and regulatory-required toxicology. It was then evaluated in Phase 0 (NCT05908773) and Phase 1 (NCT06260774) clinical trials in patients with solid tumors that indicated disease stabilization with no dose-limiting toxicities or significant safety events.

Building upon these findings, the present study extends the application of the therapeutic to the treatment of breast cancer bone metastasis, a clinically challenging and biologically distinct metastatic niche. We comprehensively evaluated MN-anti-miR10b in vitro including cellular uptake, cytotoxicity, and miR-10b silencing efficacy in parental triple-negative breast cancer cells and a bone-seeking metastatic variant. In vivo, we assessed MN-anti-miR10b containing 23-mer ASO, as well as the clinically tested TTX-MC138, for therapeutic efficacy and survival benefit in a clinically relevant mouse model of breast cancer bone colonization (hereafter referred to as “bone metastasis model” for simplicity) and showed significant survival benefits with both formulations. Collectively, these studies demonstrated that targeting miR-10b using an image-guided anti-miR-10b nanotherapeutic represents a promising and translatable strategy for targeting breast cancer bone metastases.

## 2. Materials and Methods

### 2.1. Therapeutic Synthesis and Characterization

The MN-anti-miR10b was synthesized according to the previously published protocol [36]. Briefly, a dextran-coated iron oxide-based magnetic nanoparticle (MN) core was synthesized using a co-precipitation method. Immediately after preparation, the MN was crosslinked with epichlorohydrin (Sigma Millipore, Burlington, MA, USA Cat. No. 45340) followed by reaction with ammonium hydroxide. The resultant aminated MNs, with ~26 nm hydrodynamic diameters and 70 amines per particle, were labeled with Cy5.5 by incubating with Cy5.5-NHS ester (Lumiprobe, Westminster, MD, USA Cat. No. 47020) to afford MN-Cy5.5. The number of Cy5.5 per MN particle was estimated to be 7. Next, MN-Cy5.5 was activated using N-succinimidyl 3-[2-pyridyldithio]-propionate (SPDP; Thermo Fisher Scientific, Rockford, IL, USA) and purified using a PD-10 column (Thermo Fisher Scientific, Rockford, IL, USA). In a separate tube, the 5‘-ThioMC6 end of the locked nucleic acid (LNA, Integrated DNA Technologies, Coralville, IA, USA and [36]) was activated by treating it with 3% Tris(2-carboxyethyl)phosphine hydrochloride (TCEP; Thermo Fisher Scientific, Rockford, IL, USA) followed by purification with ammonium acetate/ethanol precipitation before conjugation to the nanoparticles. Next, activated MN-Cy5.5 was incubated with activated LNA for 24 h at 4 °C under rotation to produce the MN-anti-miR10b. The number of LNA oligos per MN particle was estimated to be ~10. The hydrodynamic size, PDI and surface charge of all of the particles were measured using the dynamic light scattering method on a Zeta sizer Ultra (Malvern, Worcestershire, UK). The transmission electron microscopy image of particles was captured using high-resolution TEM (JEM-2200FS Field Emission Electron Microscope, JEOL, Tokyo, Japan) at the Center for Advanced Microscopy core of Michigan State University.

For in vivo studies, we also used TTX-MC138, an oligo-nanoparticle conjugate synthesized similar to [30] but devoid of Cy5.5 optical dye provided by TransCode Therapeutics Inc. (Woburn, MA, USA). It was synthesized with the same oligonucleotide loading efficiency as MN-anti-miR10b. All preparations were characterized and described in our previous publications [30,36].

Since the sequence specificity of the anti-miR-10b formulation has been established in our previous publications [29,30,33,37], a scrambled control formulation was not included in the present study. An additional consideration was to minimize the number of animals used, which is strongly encouraged by the National institute of Health (NIH).

### 2.2. Cell Culture

A human metastatic triple-negative breast cancer cell line expressing luciferase, MDA-MB-231-luc-D3H2LN (from there on in the manuscript, MDA-MB-231), was used in this study (Perkin Elmer/Revvity, Waltham, MA, USA). A bone-seeking subtype MDA-231-BoM-1833 was established from bone metastasis of nude mice inoculated with the parent cell line [38] and obtained from Dr. Joan Massagué (Memorial Sloan Kettering Cancer Center, New York, NY, USA). Both cell lines were cultured in DMEM (Gibco, Thermo Fisher Scientific, Rockford, IL, USA) supplemented with 10% Fetal Bovine Serum (Gibco Thermo Fisher Scientific), 100 U/mL penicillin, and 100 µg/mL streptomycin at 37 °C with 5% CO_2_ and used within the passage below 10. Cell lines were fully validated for authenticity using Short Tandem Repeat (STR) profiling (ATCC Cell Authentication Testing Service, Manassas, VA, USA) and confirmed to be free of mycoplasma contamination using PCR (Lonza MycoAlert, Lonza, Basel, Switzerland).

### 2.3. MN-Anti-miR10b Uptake Study

Uptake of MN-anti-miR10b was evaluated using both quantitative iron assay and qualitative fluorescence microscopy. For uptake analysis using iron estimation, MDA-231-BoM-1833 cells (10,000 cells/well) were plated in a 96-well plate and incubated with increasing concentrations of MN-anti-miR10b (0 to 400 µg/mL Fe) (*n* = 5–6) for two hours. Next, cells were washed with PBS and digested with 5% hydrogen peroxide in 6N hydrochloric acid for 20 min at room temperature before recording absorbance at 410 nm using a microplate reader (SpectraMax iD5, Molecular Devices, San Jose, CA, USA). The total iron amount incorporated in the cells was expressed as pg iron/cell.

For fluorescence microscopy, MDA-231-BoM-1833 cells (5 × 10^4^/well) were seeded onto glass coverslips (15 mm^2^) inserted into a 12-well plate for 48 h. MN-anti-miR10b was added to the wells at 1µM oligo concentration and incubated for 2 h. Next, the treatment medium was removed, and cells were washed with PBS and fixed with 4% paraformaldehyde before mounting with DAPI containing mounting medium (Fluoromount-G, SouthernBiotech, Birmingham, AL, USA). Cells were imaged under the fluorescence microscope (Nikon Eclipse 50i, Tokyo, Japan) at 10× magnification. All images were analyzed using Image J software version 1.53k (NIH, Bethesda, MD, USA).

### 2.4. Cell Viability Assay

The effect of MN-anti-miR10b on cell viability was measured using an MTS assay (Promega CellTiter 96^®^ AQueous One Solution, Promega, Madison, WI, USA). MDA-231-BoM-1833 or MDA-MB-231 cells were plated in a 96-well plate at 2000 cells/well in 100 µL medium. The next day, cells were treated with MN-anti-miR10b (10 µM, 1 µM, 0.1 µM and 0.01 µM oligo concentration). After 72 h of incubation, treatment medium was replaced with the MTS (Promega)-containing medium (80:20, *v*/*v*) and incubated for 1 h at 37 °C before recording the absorbance at 490 nm using a microplate reader (SpectraMax iD5, Molecular Devices, San Jose, CA, USA). The absorbance of wells without cells was used for background correction. Cell viability was represented relative to the untreated control.

### 2.5. In Vitro Apoptosis Analysis

The apoptotic effect of MN-anti-miR10b on MDA-231-BoM-1833 cells was evaluated using the Annexin V-based apoptosis assay. Briefly, 1 × 10^5^ cells per well were seeded in 6-well plates and allowed to adhere overnight. The following day, cells were treated with MN-anti-miR10b at a final oligonucleotide concentration of 1.3 µM (corresponding to the IC_50_ of MN-anti-miR10b). After 36 h, cells were harvested and stained with Annexin V-PE and 7-AAD according to the manufacturer’s instructions (Invitrogen, Carlsbad, CA, USA, Cat. No.88-8102-72). Samples were analyzed using a Cytek Aurora flow cytometer (Cytek Biosciences, Fremont, CA, USA). Flow cytometry data were processed and analyzed using FlowJo software version 10 (BD Biosciences, San Jose, CA, USA). Cell populations were classified as follows: early apoptotic cells (Annexin V^+^/7-AAD^−^), late apoptotic cells (Annexin V^+^/7-AAD^+^), and necrotic/dead cells (Annexin V^−^/7-AAD^+^). The total apoptotic fraction was defined as the combined percentage of early and late apoptotic cells.

### 2.6. Western Blot Analysis

For Western blot analysis, cell lysates were prepared 48 h after treatment with MN-anti-miR10b. Cells were lysed in RIPA buffer (Thermo Fisher Scientific) supplemented with protease inhibitor cocktail (PCI). For tumor tissue analysis, at the end of the therapeutic study, ~20 mg of harvested mouse tumor tissue was homogenized in RIPA buffer containing PCI. Protein concentrations were determined using a BCA assay (Thermo Fisher Scientific). Equal amounts of protein (30 µg per sample) were mixed with Laemmli sample buffer, denatured, and resolved on 4–20% SDS-PAGE gels. Proteins were subsequently transferred onto nitrocellulose membranes. Membranes were probed with primary antibodies against HOXD10 (LSBio, Cat. No. LS-B14549, 1:1000 dilution) and β-actin (Santa Cruz Biotechnology, Cat. No. sc-47778, 1:1000 dilution). β-actin was used as a loading control. Protein bands were visualized using enhanced chemiluminescence (ECL) detection reagent (Cytiva, Marlborough, MA, USA). Protein band intensities were quantified using ImageJ software (NIH) following densitometric analysis of Western blot images.

### 2.7. RNA Extraction and RT-qPCR Study

Total RNA from both in vitro and in vivo samples was isolated using the miRNeasy Mini Kit (Qiagen, Germantown, MD, USA), following the manufacturer’s protocols. For in vitro experiments, cells were harvested for RNA extraction 48 h after treatment at their respective IC50 concentration. For in vivo studies, RNA was extracted from ten serial tumor sections per sample, at 48 h post-injection for the biodistribution study and at the study endpoint for the therapeutic study Complementary DNA (cDNA) synthesis for miRNA analysis was performed using the miScript II RT Kit (Qiagen, Germantown, MD, USA), while quantitative real-time PCR (RT-qPCR) was carried out using the TB Green RT-qPCR Kit (Takara Bio, San Jose, CA, USA). Relative miR-10b expression levels were normalized to the U6 reference gene. Gene expression data were calculated using the comparative Ct method (2^−ΔΔCt^) and are presented as fold change relative to control samples. Primer sequences used in this study were as follows: miR-10b, 5′-TACCCTGTAGAACCG AATTTGTG-3′; U6 primers were supplied by the manufacturer.

### 2.8. Animal Model of Breast Cancer Bone Colonization

All animal experiments including pain management were conducted in accordance with ARRIVE guidelines, and the protocol (PROTO20200322) was approved by the Institutional Animal Care and Use Committee (IACUC) at Michigan State University on 18 January 2021.

Mice were housed in an AAALAC-accredited, specific pathogen-free (SPF) animal facility in individually ventilated cages with environmental enrichment. Animals had ad libitum access to food and water and were maintained on a 12 h light/dark cycle in a temperature- and humidity-controlled environment.

Bone metastasis models for both biodistribution and therapeutic efficacy studies were established in six-week-old female athymic nude mice (nu/nu; The Jackson Laboratory, Bar Harbor, ME, USA), as previously described [39]. Briefly, mice were anesthetized with 2% isoflurane and administered ketoprofen (5 mg/kg, subcutaneous) for perioperative analgesia. A small opening was created in the right tibia using a 23-gauge needle. Luciferase-expressing MDA-MB-231-BoM-1833 cells (2 × 10^5^ cells in 10 µL PBS) were then slowly injected into the tibial bone marrow cavity using an insulin syringe. Care was taken to ensure accurate placement of the cell suspension within the intramedullary space. Following intratibial tumor implantation, mice received postoperative analgesia with buprenorphine (0.05–0.1 mg/kg, subcutaneously) immediately after surgery and as needed thereafter in accordance with institutional animal care guidelines. Animals were monitored daily for signs of pain or distress throughout the study.

Tumor establishment and progression were followed by bioluminescence imaging (BLI) using a whole body IVIS imaging system (Revvity, Waltham, MA, USA). Throughout the study period, mice were housed under standard conditions with ad libitum access to food and water.

### 2.9. Biodistribution Study

Eighteen days after tumor inoculation, mice were intravenously administered either MN-anti-miR10b (10 mg Fe/kg body weight) or phosphate-buffered saline (PBS) via tail vein injection. Biodistribution and tumoral accumulation of MN-anti-miR10b were evaluated using magnetic resonance imaging (MRI, *n* = 2) and fluorescence imaging (FLI, *n* = 3) in the Cy5.5 channel. Since TTX-MN138 does not carry Cy5.5 dye, it was not used in this study.

MRI scans were acquired at baseline (pre-injection) and 24 h post-injection using a 7 Tesla BioSpec 70/30 USR system running Paravision 360v3.5, with vendor-provided coils (Bruker, Billerica, MA, USA). Mice were anesthetized using isoflurane and then were positioned prone with their legs on a 4 × 4 cm 4-channel array coil designed for rat brain imaging. An 86 mm volume coil was used as the transmit coil. To locate the tumor, an axial T2-weighted RARE scan with the following parameters was acquired: TR/TE 3082/26 ms, 4 averages, 46 slices (0.5 mm), field of view 35 × 20 mm, matrix size 175 × 100, 200 µm in-plane resolution, RARE factor 8, and scan time 2.5 min. For quantitative assessment of nanoparticle accumulation, a T2* map was acquired over the tumor using a multi-gradient echo sequence with parameters TR/TE 800/3.5 ms, 5 averages, 13 slices (0.5 mm), 50 degree flip angle, 10 positive readout echoes with 5 ms echo spacing, field of view 30 × 20 mm, matrix size 150 × 100, 200 µm in-plane resolution, and scan time 5 min. Before the T2* map acquisition, the B0 field was shimmed around the right leg using the MAPSHIM adjustment. MRI images were analyzed using the image sequence analysis module in Paravision. Regions of interest (ROIs) were drawn on 3–5 slices over the tumor or muscle on the right leg. The T2* curve was adjusted to remove longer echo times if needed to improve the fit, such that 3–10 echoes were used in the calculations. Mean T2* values for each mouse were obtained by averaging measurements across all analyzed ROIs.

At 48 h post-treatment, animals were euthanized, and major organs, including tumor-bearing tibias, were harvested for ex vivo fluorescence imaging, histological analysis, and RNA extraction to assess miR-10b inhibition. To achieve statistical significance of the data presented in Figure 3B below, we pooled mice from independent experiments that included an identical single-injection, 48 h collection protocol at the same post-implantation time point. This included the *n* = 3 MN-anti-miR10b-treated mice from the biodistribution study described above (which additionally underwent MRI/FLI imaging prior to tissue collection), together with additional mice from parallel cohorts that received the same treatment but did not undergo imaging. This resulted in a PBS control group (*n* = 5) and an MN-anti-miR10b treatment group (*n* = 6).

### 2.10. In Vivo Therapeutic Efficacy and Survival Study

For therapeutic studies, animals were inoculated with tumor cells as described above. Prior to treatment, mice were randomly divided into three groups based on equivalent tumor volumes to ensure balanced baseline tumor burden by the investigator blind to the goals of the study. Seven days following tumor implantation, treatment was started via tail vein injection. The treatment groups were: (a) PBS control (*n* = 6), (b) MN-anti-miR10b (10 mg/kg iron, *n* = 7), and (c) TTX-MC138 (10 mg/kg iron, *n* = 7). Mice received one intravenous injection per week. Treatment was continued for 8–10 weeks or until the animals became moribund.

Particle distribution and tumor growth were monitored weekly using fluorescence (FLI) and bioluminescence imaging (BLI) with an IVIS imaging system. For survival analysis, mice were euthanized upon reaching humane endpoints (inability to access food or >20% body weight loss). Survival curves were generated using Kaplan–Meier analysis, and statistical significance was determined by the log-rank test using GraphPad Prism 10. At the endpoint, tumors were collected and stored for subsequent histological and RNA analyses.

### 2.11. Immunofluorescence Analysis

Mouse tibiae, either with tumors (right tibia) or without tumors (left tibia), were collected at the experimental endpoint. Tissues were first fixed in 10% formalin for 2 days at 4 °C, followed by decalcification in 14% EDTA (pH 7.4) for 2 weeks, with the EDTA solution replaced every 3 days. After decalcification, tibiae were embedded in Jung tissue freezing medium (Leica Microsystems, Wetzlar, Germany) and cryo-sectioned into 10 μm thick sections using a Leica CM1850 cryostat (Leica Microsystems, Wetzlar, Germany).

For particle accumulation analysis, tissue sections were mounted using DAPI-containing mounting medium (Fluoromount-G, SouthernBiotech, Birmingham, AL, USA) and imaged using a fluorescence microscope (Nikon Eclipse 50i, Tokyo, Japan). Apoptotic cell death within tumor tissues was evaluated using the TUNEL assay. TUNEL staining on a 10 µm thick frozen section was carried out using a commercially available apoptosis detection kit (Cell Signaling Technology, Danvers, MA, USA, Cat. No. 48513) following the manufacturer-recommended protocol. After completion of the labeling reaction, tissue sections were mounted using a DAPI-containing mounting medium. Fluorescent images were acquired using a ZEISS Axioscan 7 (Carl Zeiss Microscopy GmbH, Jena, Germany) whole-slide scanner under 20× objective magnification. Quantitative analysis of TUNEL-positive nuclei was performed using QuPath image analysis software version 0.6.0 (University of Edinburgh, Edinburgh, UK; RRID:SCR_018257). TUNEL-positive nuclei were identified and quantified, and apoptotic amounts were calculated as the percentage of TUNEL-positive cells relative to the total number of DAPI-stained nuclei within the analyzed tumor regions.

### 2.12. Statistical Analysis

**Figure 1:**
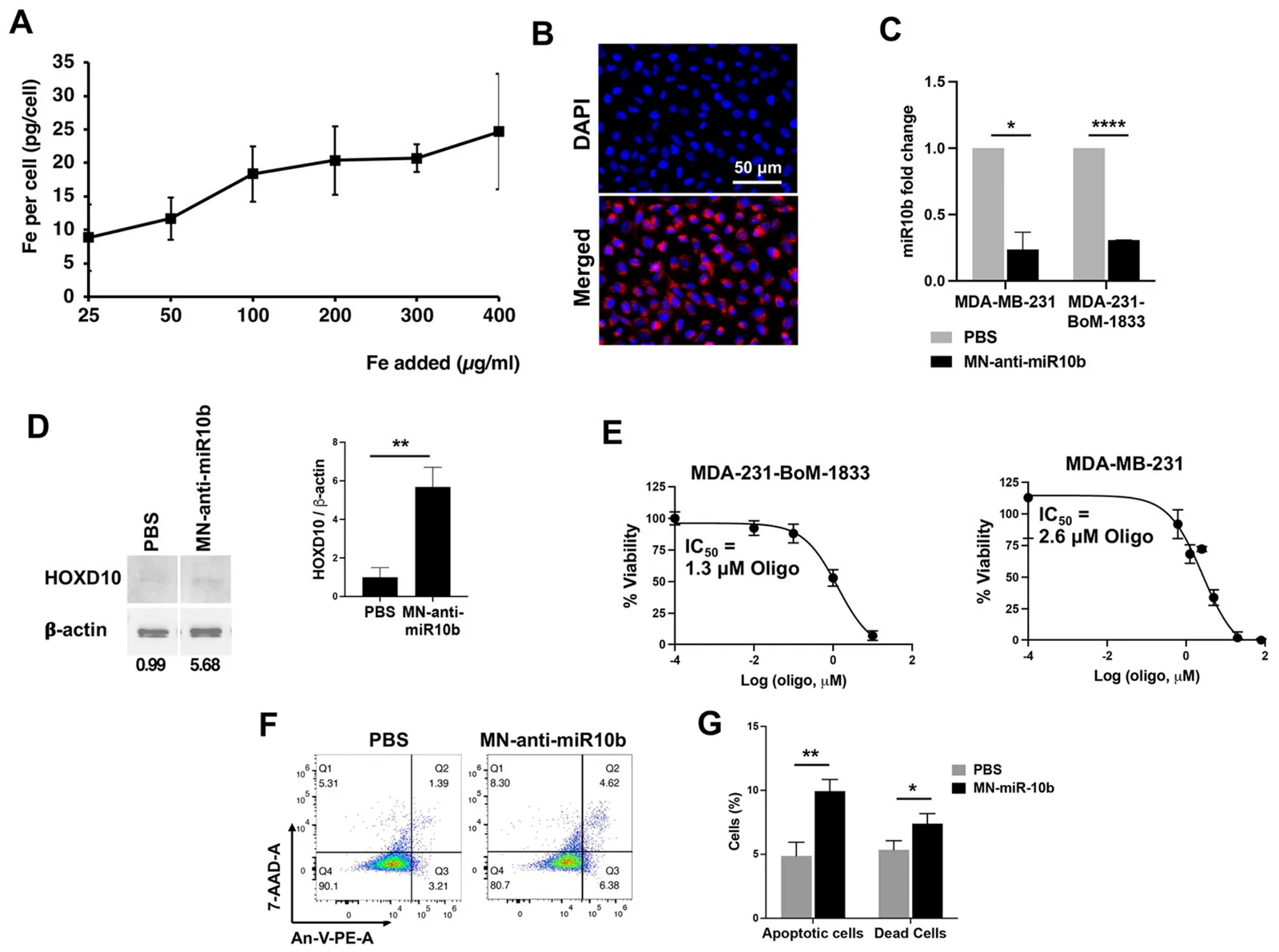
In vitro uptake and therapeutic efficacy of MN-anti-miR10b. (**A**) Quantitation of MN-anti-miR10b uptake was performed 2 h after incubation (*n* = 6). (**B**) Fluorescence microscopy depicting uptake of Cy5.5-labeled MN-anti-miR10b by MDA-231-BoM-1833 cells in (**A**). Nuclei are stained with DAPI (blue), and MN-anti-miR10b is visualized via Cy5.5 fluorescence (red). (**C**) Relative miR-10b expression levels in MDA-MB-231 and MDA-231-BoM-1833 cells 48 h after treatment with MN-anti-miR10b, as determined by RT-qPCR (*n* = 6). (**D**) Western blot analysis of HOXD10 protein levels following MN-anti-miR10b treatment showing densitometry ratios of HOXD10 to β-actin band intensity (**left**); quantitative presentation of the of HOXD10 to β-actin band intensities (**right**) (*n* = 3). The uncropped blots are shown in Supplementary Materials. (**E**) Effects of MN-anti-miR10b on cell viability of (**left**) MDA-231-BoM-1833 and (**right**) MDA-MB-231 cells following 72 h incubation, measured by MTS assay (*n* = 5). (**F**) Flow cytometry dot plots representing apoptosis in MDA-231-BoM-1833 cells following 36 h of treatment (*n* = 3). (**G**) Quantitative presentation of apoptotic cells (combined early and late apoptosis) following treatment. Statistical analysis for panels (**C**,**D**,**G**) was performed using an unpaired *t*-test (* *p* < 0.05; ** *p* < 0.01; **** *p* < 0.0001).

Data are presented as mean ± SD. Statistical analyses were performed using GraphPad Prism 10 (GraphPad Software, San Diego, CA, USA). Comparisons between two groups were analyzed using an unpaired Student’s *t*-test (Figure 1C,D,G and Figure 3B). Comparisons among groups were analyzed by one-way ANOVA followed by Tukey’s post hoc test (Figure 4C) or two-way ANOVA followed by Tukey’s post hoc test (Figure 4A). Survival was analyzed by the Kaplan–Meier method; the log-rank (Mantel–Cox) test was used to compare survival curves across all three groups with prespecified comparisons against PBS (Figure 4B). *p* values less than 0.05 were considered statistically significant.

## 3. Results

### 3.1. Preparation and Physicochemical Characterization of MN-Anti-miR10b

Dextran-coated iron oxide nanoparticles were synthesized by co-precipitation and followed by functionalization with anti-miR-10b antisense oligonucleotides, along with labelling using Cy5.5 fluorophores as described previously [36] (Figure S1A). High-resolution transmission electron microscopy (HR-TEM) confirmed the presence of uniformly distributed iron oxide cores with an average core diameter of approximately 5.7 nm (Figure S1B). Dynamic light scattering analysis showed that MN-anti-miR10b exhibited hydrodynamic diameters of 37.9 nm with a corresponding zeta potential of −26.6 mV (Figure S1C,D). The nanodrug formulation displayed low polydispersity indices (0.1–0.2), indicating a narrow size distribution and monodisperse population. The nanoparticles remained colloidally stable at 4 °C for several months, as evidenced by minimal changes in hydrodynamic diameter over time.

### 3.2. In Vitro Uptake and Functional Efficacy of MN-Anti-miR10b in Bone-Seeking Breast Cancer Cells

Following successful preparation of the MN-anti-miR10b nanodrug, its cellular uptake and functional efficacy were evaluated in the bone-seeking variant of triple-negative breast cancer cells, MDA-231-BoM-1833. In our previous work, MN-anti-miR10b was shown to effectively suppress tumor growth in lymph node metastases derived from MDA-MB-231 breast cancer in a mouse model [29]. Given the strong clinical tendency of triple-negative breast cancer to metastasize to bone, we next investigated the therapeutic potential of MN-anti-miR10b in this context.

To quantitatively validate cellular uptake, intracellular iron content was measured following 2 h exposure to MN-anti-miR10b. Treatment with increasing concentration preparation resulted in a significant increase in intracellular iron levels, rising from approximately 8.8 pg/cell to approximately 24.7 pg/cell (Figure 1A). At some point, further increases in the added therapeutic reached a plateau, suggesting saturation of nanoparticle internalization pathways. Fluorescence microscopy analysis demonstrated robust uptake of Cy5.5-labeled MN-anti-miR10b within 2 h of incubation, with most cells exhibiting strong intracellular Cy5.5 fluorescence (Figure 1B), indicating rapid and efficient nanoparticle uptake.

After confirming the efficient uptake, we next tested the ability of MN-anti-miR10b to inhibit miR-10b expression. Quantitative RT-qPCR analysis showed that treatment with MN-anti-miR10b for 48 h resulted in a significant downregulation of miR-10b expression, with a significant reduction compared to PBS-treated cells in both MDA-MB-231 (77%, * *p* < 0.05) and MDA-231-BoM-1833 (70%, **** *p* < 0.0001) cell lines (Figure 1C). To confirm the functional downstream effects of miR-10b inhibition, we evaluated the protein expression of HOXD10, a direct translational target negatively regulated by miR-10b [15]. Western blot analysis revealed that 48 h treatment with MN-anti-miR10b markedly increased HOXD10 protein expression in MDA-231-BoM-1833 cells relative to PBS (Figure 1D with original blots shown in Figure S1E), similar to what we previously observed in parental MDA-MB-231 cells [29], confirming effective miR10b pathway modulation.

We then evaluated the impact of MN-anti-miR10b on cell viability and found that the therapeutic induced a concentration-dependent reduction in cell viability in both cell lines. The half-maximal inhibitory concentration (IC_50_), expressed as oligonucleotide-equivalent dose, was determined to be ~1.3 µM in MDA-231-BoM-1833 cells (Figure 1E, left) and ~2.6 µM in parental MDA-MB-231 cells (Figure 1E, right).

Finally, we investigated whether reduced viability was associated with induction of apoptosis. Annexin V-based flow cytometric analysis demonstrated that treatment with MN-anti-miR10b significantly increased both early and late apoptotic populations, as well as necrotic/dead cells, compared to PBS-treated groups (Figure 1F,G).

Collectively, these results demonstrate that MN-anti-miR10b is efficiently internalized by bone-seeking metastatic breast cancer cells similar to the parental cell line, effectively suppresses miR-10b expression and restores HOXD10 signaling, and induces apoptosis, leading to reduced cell viability.

### 3.3. In Vivo Biodistribution and Tumor-Specific Accumulation of MN-Anti-miR10b in Bone Metastases

**Figure 2:**
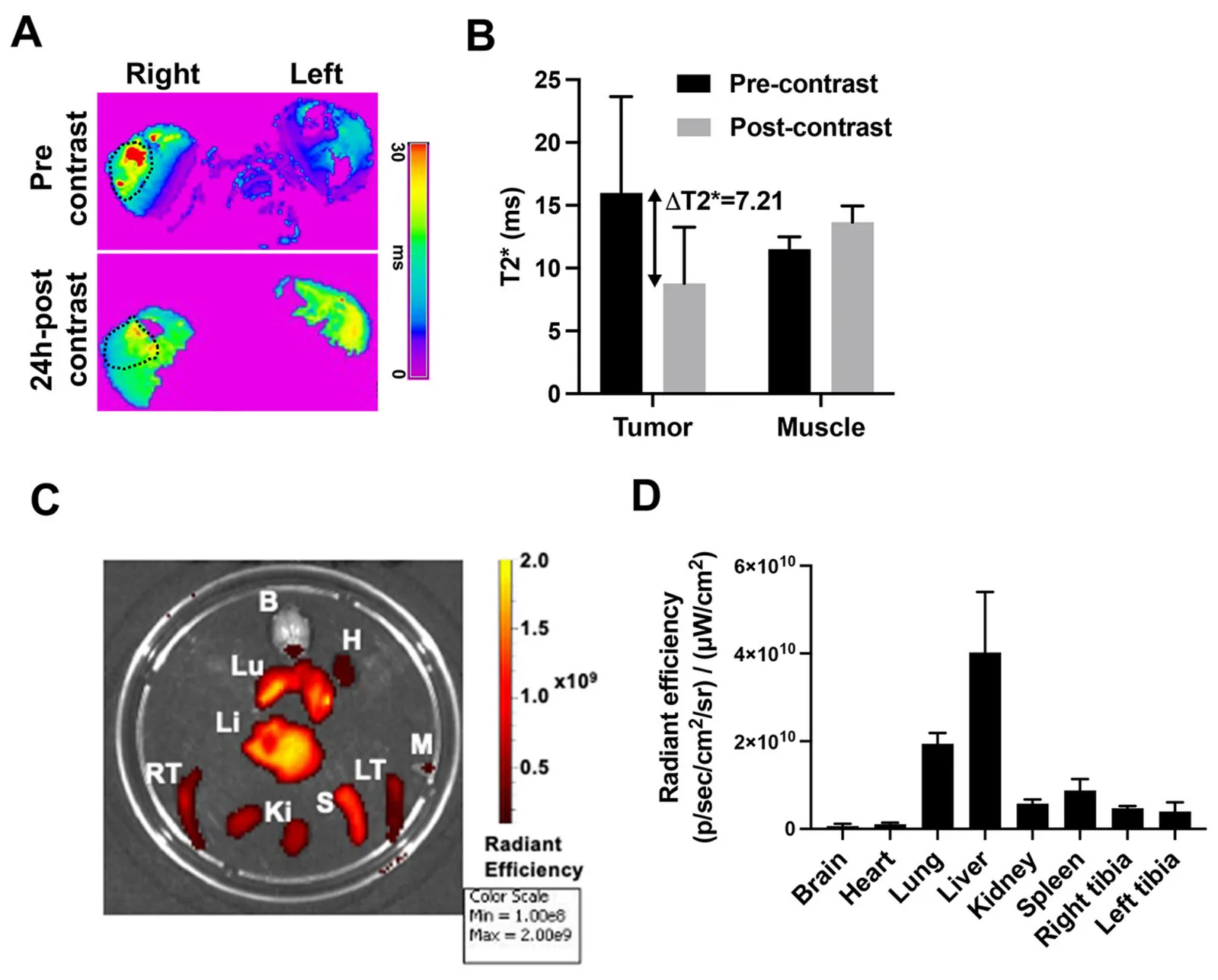
In vivo accumulation of MN-anti-miR10b in breast cancer bone metastasis. (**A**) Representative MRI T2*-map of a mouse tibia acquired pre- and 24 h post-MN-anti-miR10b administration. The right tibia shows localized signal reduction, indicating iron oxide nanoparticle accumulation. Black dotted lines represent tumor region. (**B**) Quantitative change in T2* relaxation times at 24 h post-treatment (*n* = 2). (**C**) Representative ex vivo fluorescence imaging (FLI) of major organs, including right and left tibias, harvested 48 h following MN-anti-miR10b administration. Abbreviations: B, brain; H, heart; Lu, lung; Li, liver; RT, right tibia; LT, left tibia; Ki, kidneys; S, spleen; M, muscle. (**D**) Ex vivo quantification of fluorescence radiant intensity in major organs, including bilateral tibiae, 48 h following MN-anti-miR-10b administration (*n* = 3).

Next, we investigated the in vivo biodistribution of the therapeutic and its tumor-accumulating capability in a mouse model of breast cancer bone metastasis after systemic administration. Eighteen days after tumor implantation, mice bearing tibial tumors were intravenously administered MN-anti-miR10b at a dose of 10 mg Fe/kg. T2*-weighted MRI analysis showed a marked reduction in signal intensity 24 h after nanodrug injection in the tumor-bearing right tibia compared with baseline scans, indicating localized accumulation of the iron oxide nanoparticle within the bone tumor microenvironment (Figure 2A). In contrast, no signal reduction was observed in the contralateral, non-tumor-bearing left tibia, suggesting preferential localization of MN-anti-miR10b to tumor tissue. Although these studies were performed in a small cohort, all animals demonstrated a consistent and substantial reduction in tumor T2* following MN-anti-miR10b administration (mean ΔT2* = 7.21 ms), while muscle T2* remained relatively unchanged. Indeed, quantitative analysis further demonstrated an approximately 50% decrease in T2* relaxation times in the tumor-bearing tibia at 24 h post-treatment relative to baseline values (Figure 2B). Detailed data on individual animals are presented in Figure S2A,B. These findings provide encouraging evidence of tumor-selective therapeutic accumulation and support its further evaluation.

To evaluate biodistribution to other organs, mice were euthanized 48 h following administration of the therapeutic, and major organs, including both tibias, were collected for ex vivo fluorescence imaging (FLI). As expected, MN-anti-miR10b was detected in multiple organs, with the highest accumulation observed in the liver and lungs (Figure 2C,D), consistent with clearance patterns of nanoparticle-based systems. Notably, the tumor-bearing right tibia exhibited a slightly higher fluorescence intensity compared to the non-tumor-bearing left tibia, although the contrast between tibias was less pronounced in ex vivo imaging than that observed by MRI.

**Figure 3:**
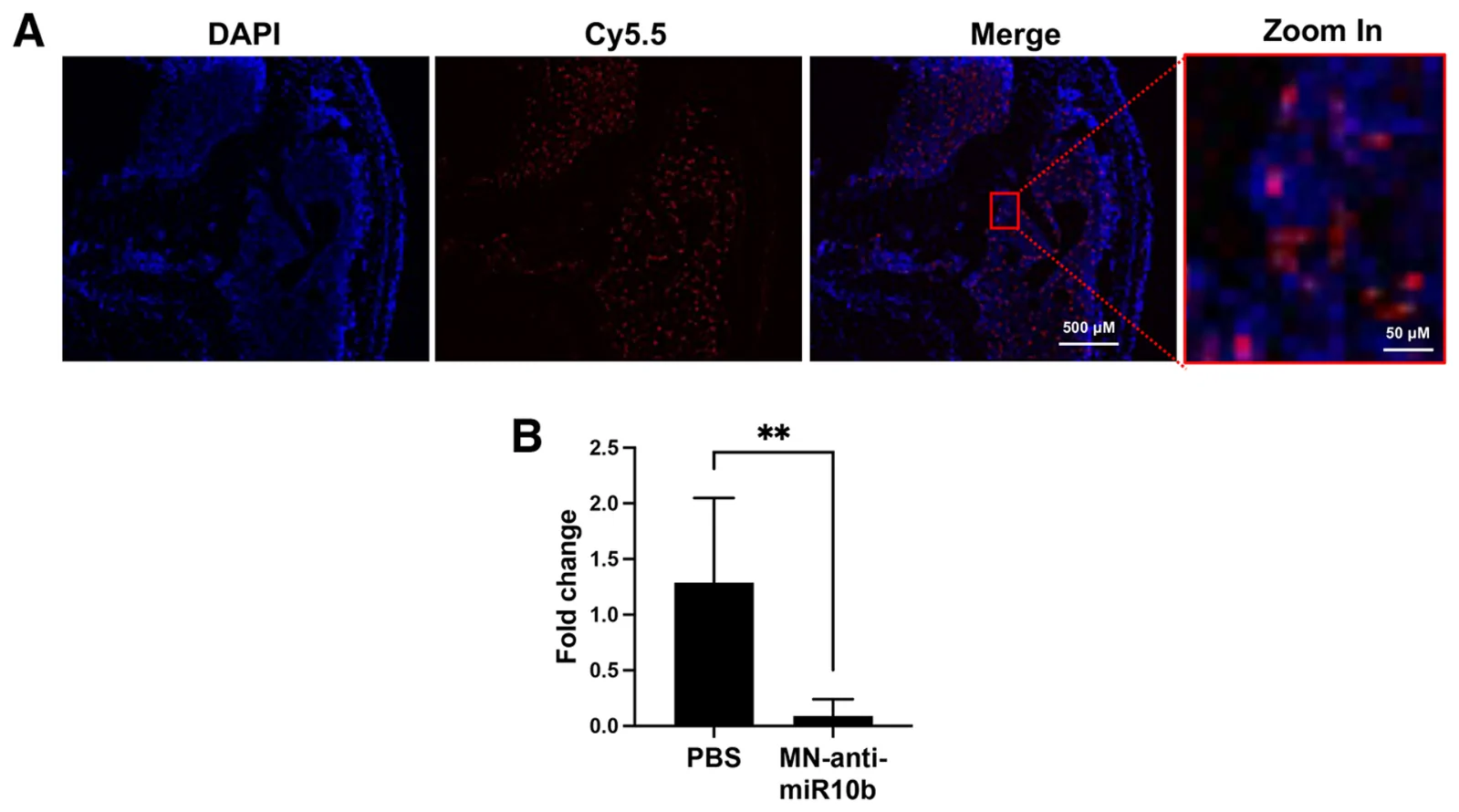
(**A**) Representative fluorescence microscopy of tumor-bearing tibial sections demonstrating accumulation of Cy5.5-labeled MN-anti-miR10b (red). Blue color represents DAPI stained nuclei. (**B**) Relative miR-10b expression levels in tumor-bearing tibias 48 h after treatment with MN-anti-miR10b, as determined by RT-qPCR (PBS, *n* = 5; MN-anti-miR10b, *n* = 6). Unpaired *t*-test. **, *p* < 0.01.

Histological examination of tumor-bearing tibial sections confirmed substantial intratumoral accumulation of Cy5.5-labeled MN-anti-miR10b, with substantial fluorescence signal detected within the bone tumor regions (Figure 3A). Importantly, this accumulation led to effective target engagement in vivo, with a significant reduction in miR-10b expression in tumor tissue after just a single injection (Figure 3B). Collectively, these findings demonstrate that systemically administered MN-anti-miR10b accumulates within breast cancer bone metastases which can be visualized using in vivo imaging.

### 3.4. Therapeutic Efficacy of Anti-miR-10b Therapeutics in a Breast Cancer Bone Colonization Model

**Figure 4:**
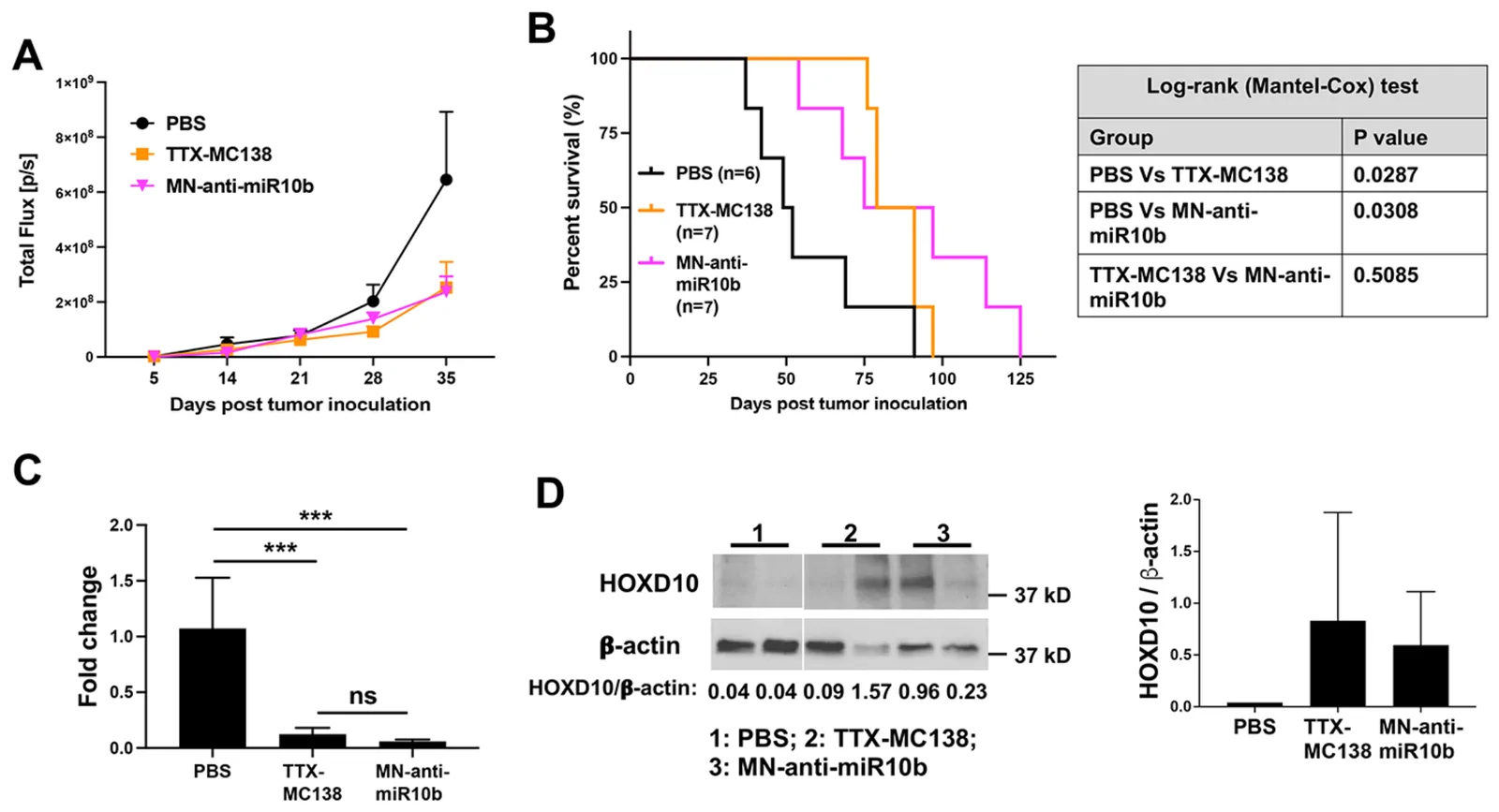
In vivo therapeutic efficacy of MN-anti-miR10b in breast cancer bone colonization model. (**A**) Tumor growth comparison among treatment groups, measured by bioluminescence imaging (BLI) (*n* = 6 in PBS group and *n* = 7 in MN-anti-miR10b and TTX-MC138 groups each). (**B**) Kaplan–Meier survival analysis. Median survival (days) compared to PBS control: PBS, 50; TTX-MC138, 85; MN-anti-miR10b, 86. Log-rank (Mantel–Cox) test across all groups, χ^2^ = 7.828, df = 2, *p* = 0.0200. (**C**) Expression level of miR-10b in tumors at the end of the study (*n* = 5/group). One-way ANOVA followed by Tukey’s multiple comparisons test was done for statistical analysis. *** *p* < 0.001; ns = not significant. (**D**) Western blot analysis showing HOXD10 upregulation. Two tumor samples from each group were analyzed. Densitometry ratio of HOXD10 to β-actin band intensity is presented below the blot (**left**), with quantification presented alongside as a bar graph (**right**) (*n* = 2/group). The uncropped blots are shown in Supplementary Materials.

After confirmation of tumor-specific accumulation of MN-anti-miR10b within tibial tumors, we next evaluated its therapeutic efficacy in a mouse model of breast cancer bone metastasis. Treatment was started seven days after intratibial tumor implantation. Mice were injected with weekly intravenous injections of PBS, MN-anti-miR10b or TTX-MC138 (a clinical-stage nanodrug therapeutic undergoing Phase I clinical evaluation for solid tumors). Analysis of tumor burden assessed by BLI showed a trend towards reduced tumor growth in mice treated with MN-anti-miR10b or TTX-MC138 compared with PBS-treated controls (Figure 4A and Figure S3A). Although BLI signal was numerically lower in the MN-anti-miR10b and TTX-MC138 groups relative to PBS at day 35, this difference did not reach statistical significance (two-way ANOVA with Tukey’s multiple comparisons test applied to Figure 4A and Figure S3A). This result is attributed to the fact that surface-weighted modalities, such as BLI, do not allow for precise volumetric measurement of the tumor due to light scatter and tissue attenuation. In addition, progressive mortality in the PBS group beyond day 35 also reduced the number of animals available for comparison at later timepoints. Kaplan–Meier survival analysis revealed a significant overall difference in survival among the three treatment groups (log-rank Mantel–Cox test, χ^2^ = 7.828, df = 2, *p* = 0.0200), with median survival of 50.0 days for PBS-treated mice compared to 85.0 days for TTX-MC138 and 86.0 days for MN-anti-miR10b (Figure 4B). Pairwise comparisons against PBS were significant for both TTX-MC138 (χ^2^ = 4.788, *p* = 0.0287) and MN-anti-miR10b (χ^2^ = 4.662, *p* = 0.0308). Importantly, repeated dosing was well tolerated with no adverse events noted. Body weight remained stable across treatment groups, comparable to PBS-treated mice (Figure S3B), indicating no apparent systemic toxicity. In vivo fluorescence imaging (FLI), based on Cy5.5 labeling of MN-anti-miR10b, demonstrated increased accumulation of the therapeutics in the tumor-bearing right tibia relative to the contralateral left tibia as tumors grew (Figure S3C), consistent with tumor-associated nanoparticle retention. By the end of the treatment (after day 63) we observed the trend in reduction of the RT/LT ratio possibly due to the reduction in tumor burden and/or saturation of the tumor with the nanoparticles.

Molecular analysis of tumor tissue collected at the study endpoints further confirmed effective target engagement. Both MN-anti-miR10b and TTX-MC138 treatment groups exhibited significant downregulation of miR-10b expression compared with PBS-treated mice (Figure 4C). Consistent with miR-10b inhibition, HOXD10 protein expression was increased in tumors from MN-anti-miR10b- and TTX-MC138-treated mice relative to the PBS control group though no formal statistical testing was performed due to the small sample size (Figure 4D with original blots shown in Figure S3D).

Tumor tissues collected at the study endpoint were analyzed for apoptosis using the TUNEL assay. TUNEL staining showed an increased number of apoptotic nuclei in MN-anti-miR10b-treated tumors compared to PBS even though this difference did not reach statistical significance (Figure S4A,B). This likely reflects the advanced stage of tumors at the endpoint, which are generally characterized by extensive necrosis that may have obscured treatment-associated apoptotic signals. Collectively, these findings demonstrate that systemic inhibition of miR-10b using an anti-miR-10b inhibition approach significantly prolongs survival and effectively engages with its target in a clinically relevant model of breast cancer bone metastasis, without evident systemic toxicity.

## 4. Discussion

Breast cancer bone metastasis represents a clinically challenging stage of disease marked by skeletal-related complications, therapeutic resistance, and limited survival [6,7]. Despite advances in systemic therapies, therapeutic options that directly target the molecular drivers of metastatic colonization are limited. In this study, we demonstrate that systemic inhibition of miR-10b using an image-guided nanoparticle platform substantially prolongs survival and restores downstream tumor suppressor signaling in a clinically relevant model of breast cancer bone metastasis. These results build upon our previous research on miR-10b inhibition by extending its application to the bone metastatic microenvironment and provide evidence that metastatic progression within bone remains dependent, at least in part, on regulatory pathways controlled by miR-10b.

MiR-10b has been increasingly recognized as a central regulator of metastatic progression in breast cancer. It promotes invasion, migration, and survival of metastatic cells through repression of tumor suppressors such as HOXD10 [15]. Our previous reports established that inhibition of miR-10b suppresses metastatic growth in lymph nodes and lungs [29,30,37], highlighting its specific role in metastatic competence. Extending these findings to the bone metastatic niche is particularly relevant, given the unique microenvironmental constraints of bone. Our data support the idea that this dependency persists after tumor cells colonize bone. Sensitivity of bone-seeking MDA-231-BoM-1833 cells to miR-10b inhibition (Figure 1C–E) is similar to what was observed with other metastatic models [29,30,37]. The continuing dependency of metastatic cells on miR-10b for survival aligns with emerging models, in which disseminated tumor cells become selectively dependent on regulatory circuits after colonization that facilitate survival in foreign microenvironments [30,40,41]. Consistent with this mechanism, inhibition of miR-10b resulted in a statistically significant increase in HOXD10 expression in vitro (Figure 1D) and in vivo even though no formal statistical testing was performed for in vivo samples due to the small sample size (Figure 4D), suggesting reactivation of HOXD10-regulated pathways that limit cellular motility and invasive behavior.

A major translational barrier for miRNA therapeutics has been systemic delivery. The iron oxide nanoparticle platform used here integrates nucleic acid stabilization, tumor-selective accumulation, and noninvasive imaging capability. Importantly, MRI-based tracking demonstrated appreciable localization of MN-anti-miR10b within tumor-bearing tibias with inhibition of miR-10b in the tibial tumor (Figure 2A,B and Figure 3B, respectively), confirming that effective target engagement in bone lesions is achievable despite the structural and vascular constraints of the bone microenvironment. In addition, the ability to noninvasively monitor delivery represents a significant advantage of our platform over oligonucleotide platforms that lack imaging capability that may help with dose optimization and patient selection in future clinical applications. While, in this study, TTX-MC138 was used for therapeutic experiments only, we have previously shown its accumulation in tumors confirmed by histology [33].

Analysis showed that survival was significantly prolonged with both MN-anti-miR10b and TTX-MC138 treatments (Figure 4A,B). This observation suggests that miR-10b inhibition may primarily delay lethal progression and metastatic deterioration in addition to inducing tumor growth inhibition. Notably, repeated dosing was well tolerated, and no systemic toxicity was observed, supporting the safety profile of this approach. These findings underscore the safety of the therapeutic (notably, TTX-MC138), which has already been tested in clinical trials with preliminary evidence of disease stabilization and no dose-limiting toxicities or significant safety events.

Further optimization of nanoparticle delivery strategies may enhance the therapeutic potential of this platform. As with many systemically administered nanoparticles, a portion of the injected therapeutic dose accumulates in organs such as the liver and lung, which mediate their systemic clearance. While this observation is in line with our previously reported biodistribution data [42] it also points to an opportunity for continued refinement of nanoparticle surface chemistry or incorporation of bone-targeting moieties to further improve tumor specificity. In addition, while the present study focused on triple-negative breast cancer, extending this approach to other molecular subtypes, including hormone receptor-positive and HER2-positive breast cancer bone metastasis models, would help determine the broader applicability of miR-10b inhibition.

We also need to acknowledge that the intratibial injection used here is a model of bone colonization/tumor growth within the bone, and not the complete metastatic cascade. We are aware that this model bypasses the initial stages of the metastatic cascade such as primary tumor growth, invasion, intravasation, systemic circulation, and homing to the bone. At the same time, the majority of patients are diagnosed at a late stage of bone lesion progression, often only after symptoms or complications appear. Consequentially, in this study, our objective was not to model the full systemic metastatic process, but rather to evaluate the efficacy of our therapeutic on intraosseous tumor growth. Importantly, the intratibial model, widely used in research, was selected because it provides a highly reproducible, synchronized, and site-specific tumor burden within the bone compartment, allowing us to accurately measure local changes without confounding variations in metastatic homing efficiency. Additional mechanistic studies investigating how miR-10b regulates tumor–bone interactions may provide deeper insight into the molecular dependencies that support metastatic growth within the bone microenvironment.

Current management of breast cancer bone metastases relies on a combination of systemic anticancer therapy, bone-modifying agents, and local interventions when clinically indicated. Bone-modifying agents, including denosumab and bisphosphonates such as zoledronic acid, are routinely recommended to reduce skeletal-related events, while radiation therapy and surgical stabilization are used for painful, structurally compromising, or neurologically threatening lesions. Importantly, these approaches primarily address tumor burden according to breast cancer subtype and/or the consequences of tumor–bone interactions and skeletal destruction, rather than specifically targeting molecular drivers of metastatic dissemination. In this context, anti-miR-10b therapy represents a mechanistically distinct therapeutic strategy aimed at directly targeting miR-10b, a molecular regulator implicated in tumor progression and metastatic behavior. Our findings suggest that clinically translatable anti-miR-10b therapy using TTX-MC138 may have potential as a systemic anticancer approach for bone metastases. It is widely recognized that the combination of two or more therapeutic treatments to specifically target carcinogenesis is a cornerstone of cancer therapy [43,44]. Rather than replacing established bone-modifying or local therapies, anti-miR-10b treatment could potentially be integrated with these approaches or with subtype-specific systemic therapies to simultaneously target tumor cells and the bone microenvironment. Future studies will be required to determine whether combining anti-miR-10b therapy with current standards of care can provide additive or synergistic therapeutic benefit and, importantly, whether such combinations can reduce tumor progression and skeletal complications more effectively than existing approaches alone.

## 5. Conclusions

In conclusion, our findings provide compelling evidence that targeting miR-10b within the bone metastatic niche is both feasible and therapeutically beneficial when coupled to a delivery system capable of protecting and guiding the oligonucleotide payload. By combining molecular targeting with image-guided delivery, our therapeutic approach represents a promising theranostic alternative for treatment of metastatic breast cancer.

## Data Availability

The data generated by the authors are on file at MSU and are included in the article.

## Supplementary Materials

The following supporting information can be downloaded at https://www.mdpi.com/article/10.3390/cancers18172731/s1: Figure S1: Physicochemical characterization of MN-anti-miR10b. (A) Schematic illustration of the MN-anti-miR10b nanodrug, consisting of an iron oxide core coated with dextran and surface-conjugated with anti-miR-10b antisense oligonucleotides and Cy5.5 fluorophores. (B) Representative high-resolution transmission electron microscopy (HR-TEM) image of MN-anti-miR10b nanoparticles; the red circle highlights an individual nanoparticle. Scale bar = 50 nm. (C) Representative hydrodynamic diameter distribution and (D) zeta potential profile of MN-anti-miR10b measured by dynamic light scattering (DLS). (E) Original uncropped Western blot for Figure 1D showing the bands with molecular markers. Lane 1 (PBS-1) and lane 3 (MN-anti-miR10b-1), indicated by the red dotted rectangle, were cropped and used in the representative Figure 1D. Unrelated lines were redacted; Figure S2: T2* values measured in tumor and muscle before (pre-contrast MRI) and after (post-contrast MRI) therapeutic administration in individual animals. (A) Individual T2* values with means. (B) Change in T2* (∆T2*) for each animal and mean ± SD; Figure S3: (A) Representative in vivo bioluminescence imaging (BLI) of mice 35 days after tumor inoculation, illustrating tumor burden in the tibial model in various groups. (B) Changes in animal body weight over the course of treatment with MN-anti-miR10b (*n* = 7) or TTX-MC138 (*n* = 7) compared to PBS-injected group (*n* = 6). (C) Ratio of total radiant efficiency between the tumor-bearing right tibia (RT) and the contralateral left tibia (LT) based on Cy5.5 fluorescence imaging (FLI) signal following treatment with MN-anti-miR10b (*n* = 7). (D) Original uncropped Western blot for Figure 4D showing the bands with molecular markers. Lanes 1 and 2 (PBS group); lanes 5 and 6 (TTX-MC138 group); and lanes 7 and 8 (MN-anti-miR10b group), indicated by the red dotted rectangles, were cropped and used in the representative Figure 4D. Unrelated lines were redacted; Figure S4: TUNEL analysis of apoptosis in right tibial tumors at study endpoint. TUNEL-positive cells were evaluated in right tibial tumor sections from mice treated with TTX-MC138, MN-anti-miR10b or PBS (*n* = 4 per group). (A) Representative TUNEL-stained images from each treatment group. Cell nuclei are stained with DAPI (blue), TUNEL-positive nuclei are labeled with CF594 (red), and merged images show the overlay of DAPI and TUNEL signals. (B) Quantitative analysis showing the percentage of TUNEL-positive nuclei relative to total DAPI-positive nuclei.

## Abbreviations

The following abbreviations are used in this manuscript:

RANKL	Receptor Activator of Nuclear Factor-κB Ligand
miR	microRNA
MRI	Magnetic Resonance Imaging
MN	Dextran-coated Iron Oxide-based Magnetic Nanoparticle
ASO	Antisense Oligonucleotide
LNA	Locked Nucleic Acid
Cy5.5-NHS	Cyanine5.5 N-hydroxysuccinimide Ester
PDI	Polydispersity Index
HR-TEM	High-resolution Transmission Electron Microscopy
DMEM	Dulbecco’s Modified Eagle Medium
PE	Phycoerythrin
7-AAD	7-aminoactinomycin D
BCA	Bicinchoninic Acid
IVIS	In Vivo Imaging System
FLI	Fluorescence Imaging
RARE	Rapid Acquisition with Relaxation Enhancement
BLI	Bioluminescence Imaging
TUNEL	Terminal deoxynucleotidyl transferase dUTP Nick End Labeling
