# Supplementary material for: Targeting miR-10b in Breast Cancer Bone Colonization Model Using Image-Guided Nucleic Acid-Based Therapeutics
Source: Cancers (Basel). 2026 Aug 23;18(17):2731. doi: 10.3390/cancers18172731 (PMC13565662; doi:10.3390/cancers18172731)
Supplement: Supplementary file 1 [file cancers-18-02731-s001.zip › cancers-4471642-supplementary.pdf]

Figure S1

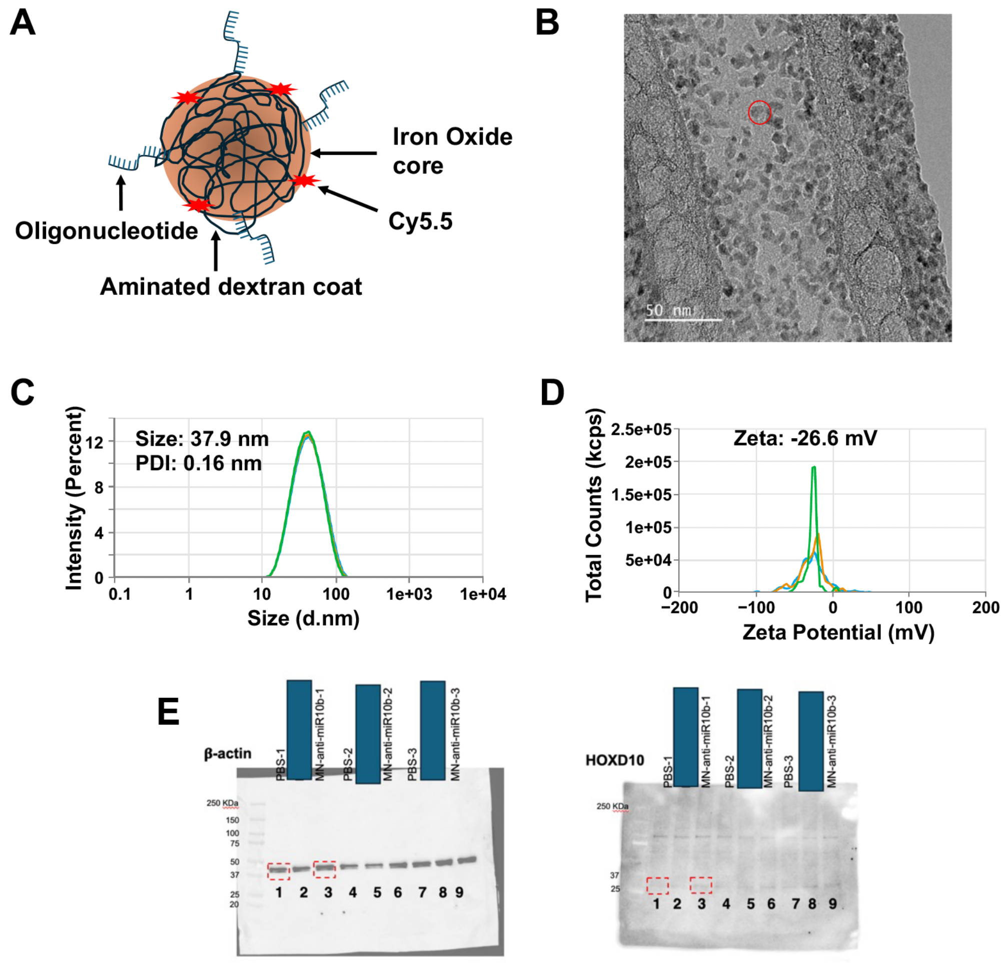
A
Iron Oxide core
Cy5.5
Oligonucleotide
Aminated dextran coat
B
50 nm
C
Size: 37.9 nm
PDI: 0.16 nm
Intensity (Percent)
0
4
8
12
0.1
1
10
100
1e+03
1e+04
Size (d.nm)
D
Zeta: -26.6 mV
Total Counts (kcps)
0
5e+04
1e+05
1.5e+05
2e+05
2.5e+05
−200
−100
0
100
200
Zeta Potential (mV)
E
β-actin
PBS-1
MN-anti-miR10b-1
PBS-2
MN-anti-miR10b-2
PBS-3
MN-anti-miR10b-3
250 KDa
150
100
75
50
37
25
20
1 2 3 4 5 6 7 8 9
HOXD10
PBS-1
MN-anti-miR10b-1
PBS-2
MN-anti-miR10b-2
PBS-3
MN-anti-miR10b-3
250 KDa
37
25
1 2 3 4 5 6 7 8 9

Figure S2

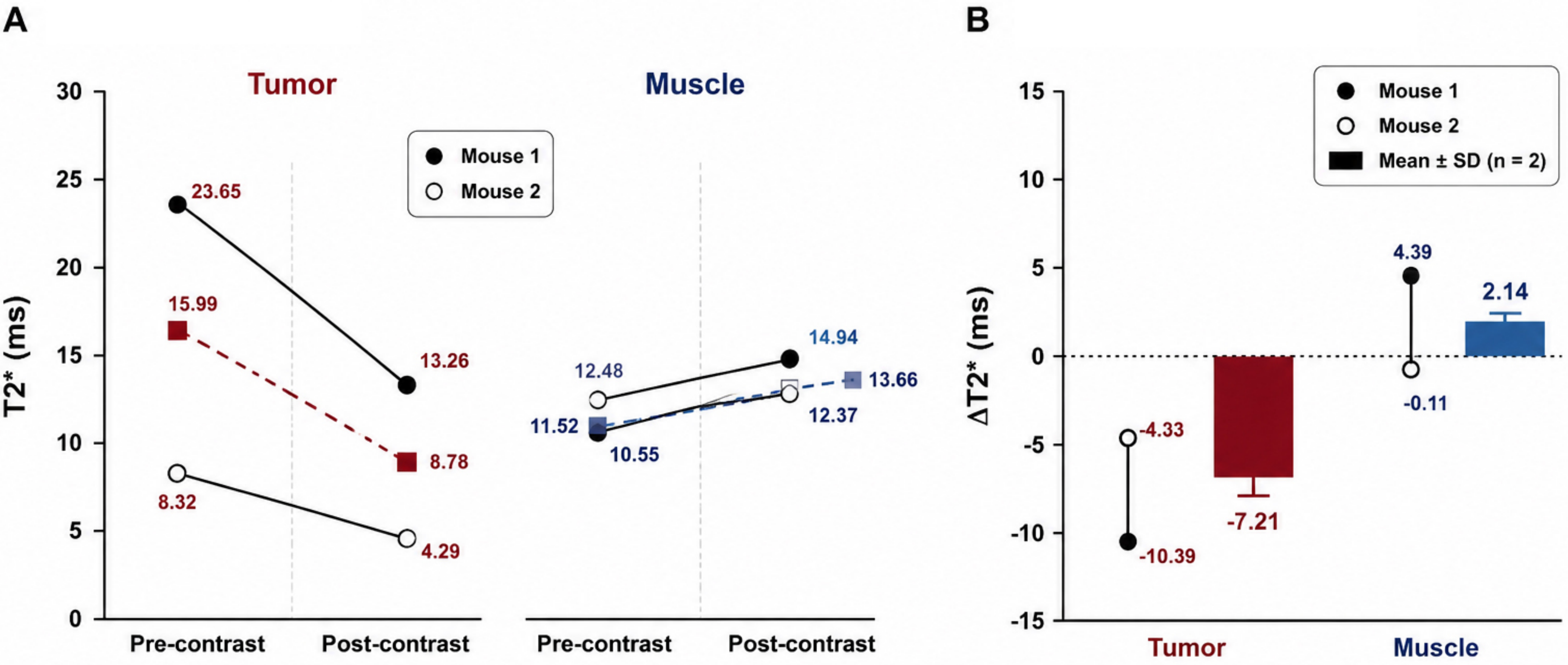

		T2* (ms)			ΔT2* (Post – Pre, ms)		
		Pre-contrast	Post-contrast	Mean ± SD	Mouse 1	Mouse 2	Mean ± SD
Tumor	Mouse 1	23.65	13.26	15.99 ± 10.83	-10.39	-4.03	-7.21 ± 4.50
	Mouse 2	8.32	4.29				
Muscle	Mouse 1	10.55	14.94	11.52 ± 1.36	4.39	-0.11	2.14 ± 3.18
	Mouse 2	12.48	12.37				

Figure S3

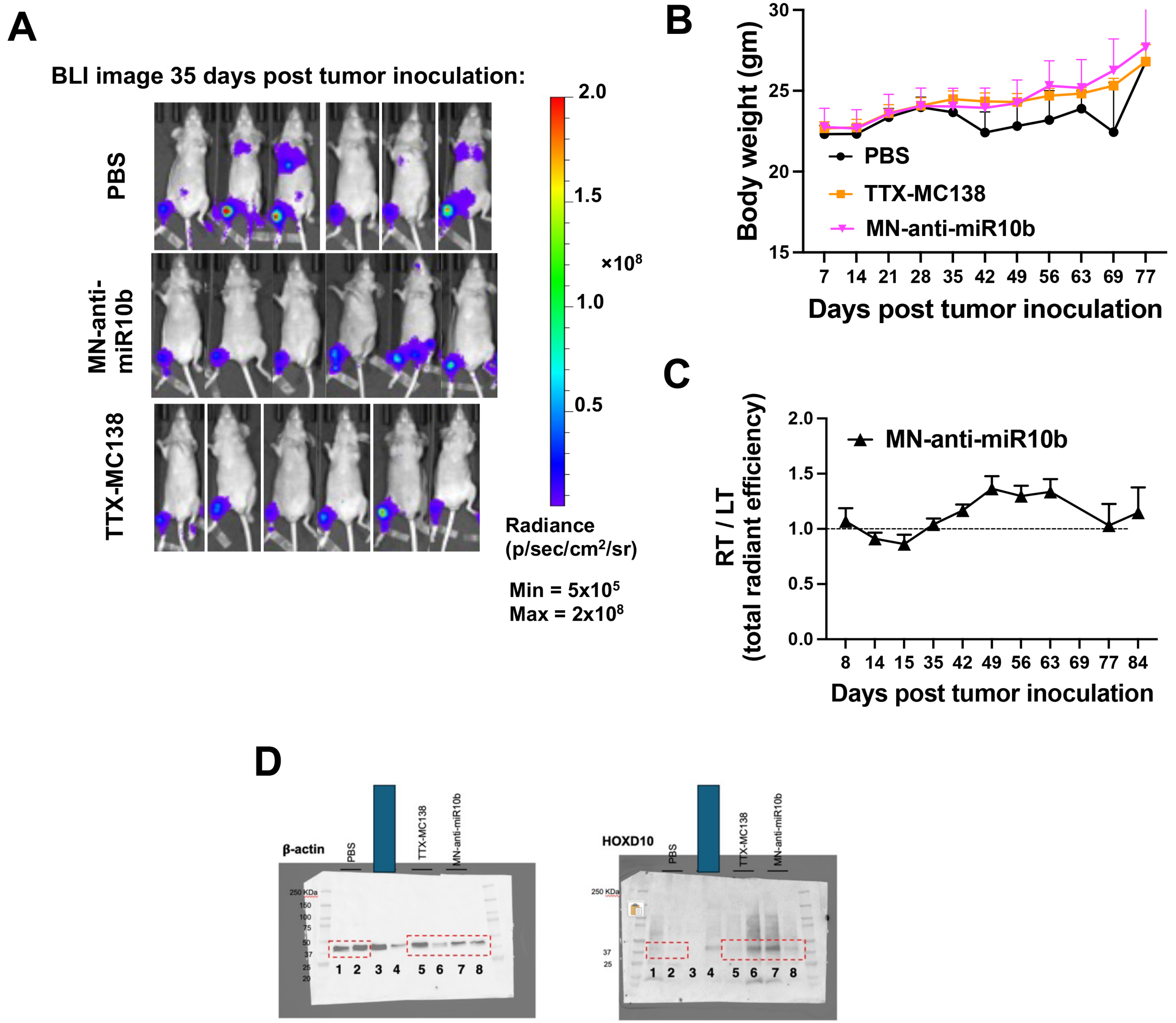
A
BLI image 35 days post tumor inoculation:
PBS
MN-anti-miR10b
TTX-MC138
2.0
1.5
×10^8
1.0
0.5
Radiance (p/sec/cm²/sr)
Min = 5x10^5
Max = 2x10^8
B
Body weight (gm)
PBS
TTX-MC138
MN-anti-miR10b
Days post tumor inoculation
C
RT / LT (total radiant efficiency)
MN-anti-miR10b
Days post tumor inoculation
D
β-actin
HOXD10
PBS
TTX-MC138
MN-anti-miR10b
250 KDa
1 2 3 4 5 6 7 8

Figure S4

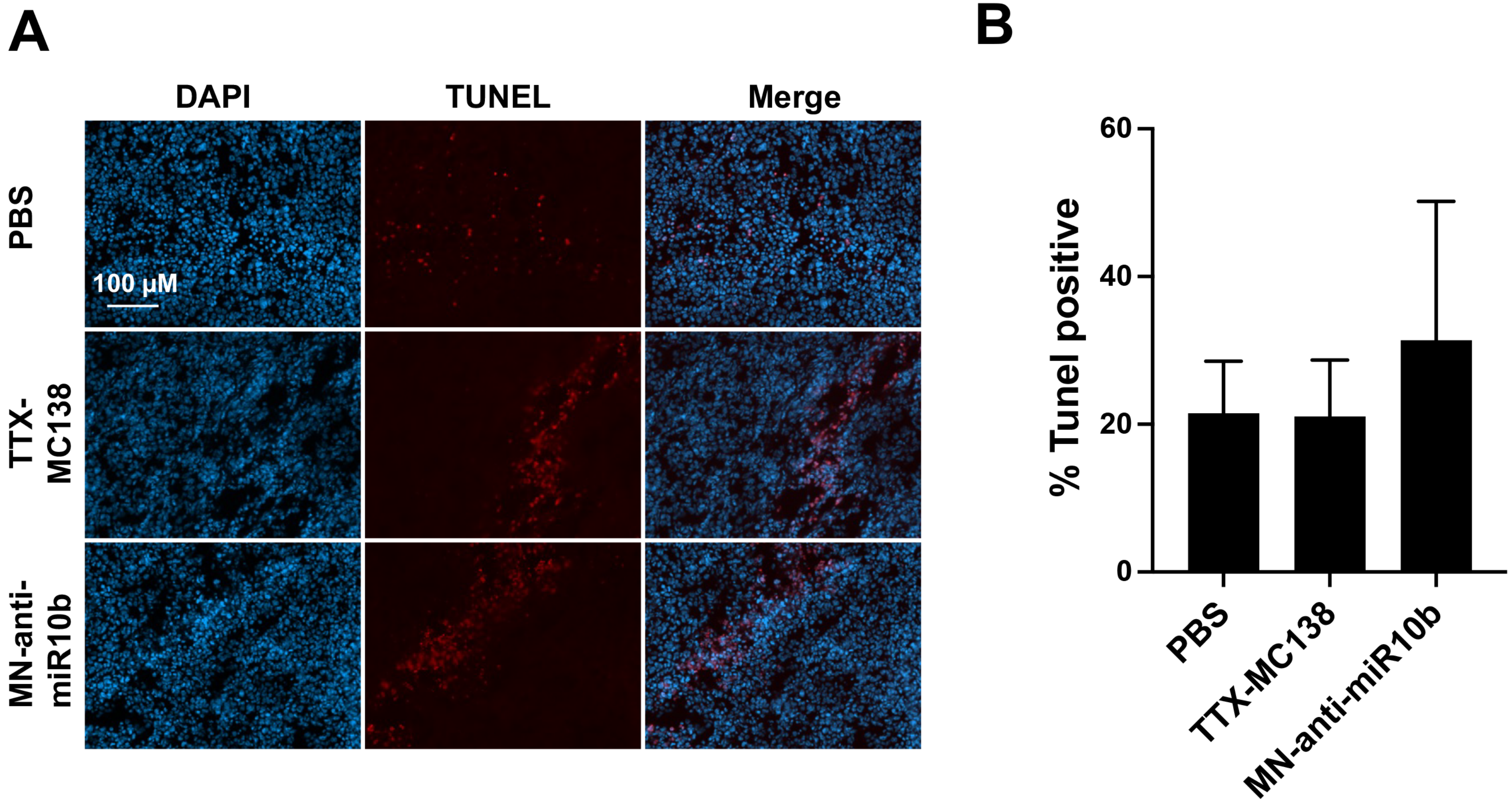
A
DAPI
TUNEL
Merge
PBS
TTX-MC138
MN-anti-miR10b
100 μM
B
% Tunel positive
0
20
40
60
PBS
TTX-MC138
MN-anti-miR10b
